# *In Vitro* Antibacterial Properties of Cefiderocol, a Novel Siderophore Cephalosporin, against Gram-Negative Bacteria

**DOI:** 10.1128/AAC.01454-17

**Published:** 2017-12-21

**Authors:** Akinobu Ito, Takafumi Sato, Merime Ota, Miki Takemura, Toru Nishikawa, Shinsuke Toba, Naoki Kohira, Satoshi Miyagawa, Naoki Ishibashi, Shuhei Matsumoto, Rio Nakamura, Masakatsu Tsuji, Yoshinori Yamano

**Affiliations:** a Shionogi & Co., Ltd., Toyonaka, Osaka, Japan

**Keywords:** cefiderocol, time kill, penicillin-binding protein, PBP, morphology, iron transporter, efflux pump, porin

## Abstract

Cefiderocol (CFDC; S-649266), a novel parenteral siderophore cephalosporin conjugated with a catechol moiety, has a characteristic antibacterial spectrum with a potent activity against a broad range of aerobic Gram-negative bacterial species, including carbapenem-resistant strains of Enterobacteriaceae and nonfermenting bacteria such as Pseudomonas aeruginosa and Acinetobacter baumannii. Cefiderocol has affinity mainly for penicillin-binding protein 3 (PBP3) of Enterobacteriaceae and nonfermenting bacteria similar to that of ceftazidime. A deficiency of the iron transporter PiuA in P. aeruginosa or both CirA and Fiu in Escherichia coli caused 16-fold increases in cefiderocol MICs, suggesting that these iron transporters contribute to the permeation of cefiderocol across the outer membrane. The deficiency of OmpK35/36 in Klebsiella pneumoniae and the overproduction of efflux pump MexA-MexB-OprM in P. aeruginosa showed no significant impact on the activity of cefiderocol.

## INTRODUCTION

Nosocomial infections caused by Gram-negative bacteria are increasingly difficult to treat due to the global spread of multidrug-resistant (MDR) strains which are resistant to several antibiotics, such as carbapenems, cephalosporins, aminoglycosides, and quinolones (1). The WHO has listed carbapenem-resistant Enterobacteriaceae (CRE), carbapenem-resistant Pseudomonas aeruginosa, and carbapenem-resistant Acinetobacter baumannii as the pathogens against which urgent development of new antibiotics are needed, since the emergence of these resistant pathogens poses serious public health issues due to the limited number of treatment options (2, 3).

Since the 1980s, numerous attempts to conjugate iron-binding functional groups onto β-lactams have been made to hijack the iron uptake systems of Gram-negative bacteria and circumvent the outer membrane barriers (4–6). However, none of the molecules have been approved for clinical use for various reasons, such as a lack of correlation between *in vitro* and *in vivo* efficacies (7–9). Cefiderocol (CFDC; S-649266), a novel catechol-substituted siderophore cephalosporin, is structurally different from other recently developed hydroxypyridone-substituted siderophore monobactam antibiotics such as BAL30072, MB-1, and MC-1 and has been reported to have potent antibacterial activity against MDR Gram-negative pathogens, including carbapenem-resistant strains of Enterobacteriaceae, P. aeruginosa, and A. baumannii, as well as potent *in vivo* efficacy against multiple clinical strains of Gram-negative bacteria in mouse lung infection models (10–14, 34; I. Ghazi, M. L. Monogue, M. Tsuji, and D. P. Nicolau, submitted for publication). This is the first report evaluating the *in vitro* features of cefiderocol, including its antibacterial spectrum against Gram-negative and Gram-positive bacteria and its mode of action, such as penicillin-binding protein (PBP) binding affinity and morphological changes, as well as the impacts of various β-lactamases, efflux pump overexpression, and deficiency of porin or iron transporter on the *in vitro* activity.

## RESULTS

### Antibacterial activity against Gram-negative and Gram-positive bacteria.

The MICs of cefiderocol were ≤2 μg/ml against a broad range of Gram-negative bacterial strains, including Enterobacteriaceae such as Enterobacter spp., Escherichia coli, Klebsiella spp., Proteus spp., Providencia spp., Salmonella spp., and Yersinia spp., as well as Vibrio species (Table 1). Cefiderocol showed *in vitro* activity against nonfermenting bacteria such as Acinetobacter spp., Pseudomonas spp., and Burkholderia spp.; cefiderocol also showed *in vitro* activity against the intrinsically MDR bacteria of Stenotrophomonas maltophilia and Elizabethkingia meningoseptica, as well as the causative pathogens for respiratory tract infections, such as Haemophilus spp., Moraxella catarrhalis, and Bordetella parapertussis. On the other hand, the MICs of cefiderocol against Campylobacter jejuni ATCC 33560 and 794009 as well as ceftriaxone-resistant Neisseria gonorrhoeae 868339 were relatively high (MIC, >4 μg/ml) for the Gram-negative bacteria tested, although the MICs of cefiderocol against two other N. gonorrhoeae strains, including an azithromycin-resistant strain, were ≤0.5 μg/ml. The MICs of cefiderocol against aerobic Gram-positive bacteria growing in an aerobic or microaerophilic atmosphere were ≥4 μg/ml, except for those against Streptococcus pneumoniae ATCC 49619, Streptococcus pyogenes ATCC 10389, and Micrococcus luteus ATCC 9341, which were 2, 1, and 4 μg/ml, respectively. The activities against these strains of Gram-positive bacteria were weaker than those of other tested β-lactam compounds. The MICs of cefiderocol against anaerobic Gram-negative and Gram-positive bacteria showed variation within genera and were higher than those of cefepime or meropenem, with cefiderocol MICs of 0.5 to >32 μg/ml, except for that against Fusobacterium necrophorum, which was ≤0.031 μg/ml (Table 2). Although cefiderocol showed activity against some ATCC strains of Bacteroides spp., Prevotella spp., and Clostridium spp., with MICs of 1 to 2 μg/ml, cefiderocol did not show potent activity against multiple clinical isolates of Bacteroides spp., Prevotella spp., or Clostridium difficile, of which the MIC_50_s were 32 μg/ml or higher (Table 3).

**TABLE 1:** MICs of cefiderocol and other antibiotics against Gram-negative and Gram-positive bacteria

Organism	Strain	MIC (μg/ml)^*f*^									
		CFDC	CAZ	CFPM	MEPM	PIPC-TAZ	CAZ-AVI	CFT-TAZ	COL	AMK	CPFX
Gram-negative bacteria											
Acinetobacter baumannii	ATCC 19606	0.063	8	16	1	16	16	2	0.5	16	1
Acinetobacter calcoaceticus	ATCC 23055	0.063	0.125	0.063	≤0.031	≤0.031	0.063	≤0.031	0.25	0.25	≤0.031
Acinetobacter haemolyticus	ATCC 17906	≤0.031	2	1	0.25	≤0.031	4	≤0.031	1	>32	0.125
Acinetobacter johnsonii	ATCC 17909	0.125	8	2	0.25	≤0.031	16	≤0.031	0.25	1	0.25
Acinetobacter lwoffii	ATCC 15309	≤0.031	1	0.25	0.063	≤0.031	2	≤0.031	0.125	0.5	0.25
Aeromonas hydrophila	IFO3820	0.125	0.5	0.063	2	4	0.25	1	>32	2	≤0.031
Bordetella parapertussis	NCTC 5952	1	1	1	≤0.031	≤0.031	0.5	1	≤0.031	2	≤0.031
Burkholderia cepacia	ATCC 25416	≤0.031	4	32	4	32	2	2	>32	>32	1
Burkholderia multivorans	SR01869	2	2	32	4	>32	2	4	>32	>32	4
Campylobacter jejuni	ATCC 33560	>4	>16	2	0.015	64	4	NT	NT	NT	0.25
Campylobacter jejuni	794009	>4	>16	2	0.015	64	4	NT	NT	NT	>4
Citrobacter freundii	ATCC 8090	0.063	1	≤0.031	≤0.031	1	0.125	0.25	0.5	2	≤0.031
Elizabethkingia meningoseptica	NCTC 10016	1	>32	>32	16	>32	>32	>32	>32	32	8
Enterobacter aerogenes	ATCC 13048	≤0.031	1	0.063	0.063	4	0.5	0.5	0.25	2	≤0.031
Enterobacter cloacae	ATCC 13047	0.5	8	0.125	0.063	16	0.5	8	>32	2	≤0.031
Enterobacter cloacae^*a*^	NCTC 13464	0.125	2	8	0.063	2	0.25	0.5	0.25	2	≤0.031
Escherichia coli	ATCC 25922	0.125	0.5	0.125	≤0.031	2	0.5	0.25	0.5	1	≤0.031
Escherichia coli	ATCC 35218	≤0.031	0.25	0.125	≤0.031	1	0.125	0.25	0.5	2	≤0.031
Escherichia coli^*a*^	NCTC 13462	2	8	>32	≤0.031	2	1	2	0.25	4	0.25
Haemophilus influenzae	ATCC 10211	0.25	0.063	≤0.031	0.063	≤0.031	≤0.031	0.125	0.25	8	≤0.031
Haemophilus influenzae	ATCC 49247	2	0.5	1	0.125	0.125	0.125	1	0.25	8	≤0.031
Haemophilus parainfluenzae	ATCC 7901	0.25	≤0.031	≤0.031	≤0.031	≤0.031	≤0.031	≤0.031	0.25	1	≤0.031
Klebsiella oxytoca	ATCC 13182	0.063	0.25	0.125	0.063	4	0.5	0.5	0.25	2	0.063
Klebsiella pneumoniae	ATCC 43816	≤0.031	0.125	0.063	0.063	2	0.25	0.25	0.25	1	0.063
Moraxella catarrhalis	ATCC 25238	≤0.031	0.063	0.125	≤0.031	≤0.031	0.063	≤0.031	1	0.5	≤0.031
Morganella morgannii	ATCC 25830	≤0.031	0.125	0.063	0.125	≤0.031	0.063	0.125	>32	1	≤0.031
Neisseria gonorrhoeae	ATCC 49226	0.5	0.12	0.12	0.03	≤0.25	0.12	NT	NT	NT	0.004
Neisseria gonorrhoeae^*b*^	867807	0.25	0.12	0.06	0.015	≤0.25	0.12	NT	NT	NT	0.008
Neisseria gonorrhoeae^*c*^	868339	>4	>16	>8	0.06	1	>16	NT	NT	NT	>4
Neisseria meningitidis	ATCC 13077	0.125	0.063	≤0.031	≤0.031	≤0.031	≤0.031	≤0.031	>32	8	≤0.031
Proteus mirabilis	ATCC 29906	≤0.031	0.25	0.125	0.125	0.5	0.125	0.5	>32	4	≤0.031
Proteus vulgaris	ATCC 13315	≤0.031	0.125	0.063	0.125	≤0.031	0.063	0.25	32	0.5	≤0.031
Providencia alcalifaciens	ATCC 9886	≤0.031	0.25	0.063	0.063	2	0.25	0.125	>32	4	≤0.031
Providencia rettgeri	ATCC 29944	≤0.031	0.25	1	0.063	4	0.25	>32	>32	0.5	≤0.031
Providencia stuartii	ATCC 29914	≤0.031	0.25	0.063	0.125	1	0.5	0.25	>32	0.5	≤0.031
Pseudomonas aeruginosa	ATCC 27853	0.5	2	2	0.25	2	2	0.5	0.5	4	0.25
Pseudomonas aeruginosa^*d*^	NCTC 13437	0.5	>32	>32	>32	>32	>32	>32	1	32	32
Pseudomonas putida	ATCC 12633	0.125	2	2	1	8	2	0.5	0.5	0.5	0.063
Pseudomonas stutzerii	ATCC 11607	0.125	0.5	0.125	0.125	2	0.5	0.125	0.125	0.5	≤0.031
Salmonella enterica serovar Choleraesuis	ATCC 51741	0.015	0.5	0.06	0.03	4	0.5	NT	NT	NT	0.03
Salmonella enterica serovar Enteritidis	G-14	0.25	1	0.063	≤0.031	2	0.5	1	1	16	≤0.031
Salmonella enterica serovar Paratyphi	598989	0.06	0.5	0.06	0.03	4	0.25	NT	NT	NT	>4
Salmonella enterica serovar Typhi	673937	0.015	0.5	0.12	0.03	2	0.25	NT	NT	NT	>4
Salmonella enterica serovar Typhimurium	ATCC 13311	≤0.031	0.5	0.063	≤0.031	4	0.5	0.5	0.5	2	≤0.031
Serratia marcescens	ATCC 13880	≤0.031	0.5	0.25	0.063	2	1	0.5	>32	1	0.063
Shigella flexneri^*e*^	705927	0.5	0.06	0.25	0.03	2	0.06	NT	NT	NT	0.015
Stenotrophomonas maltophilia	ATCC 13637	0.125	32	>32	>32	>32	32	32	1	4	0.25
Vibrio fluvialis	NCTC 11327	0.25	0.25	0.25	0.25	4	0.5	4	0.25	2	≤0.031
Vibrio vulnificus	ATCC 27562	1	0.5	1	≤0.031	0.063	≤0.031	2	4	2	≤0.031
Yersinia enterocolitica	ATCC 9610	≤0.031	0.125	0.063	0.063	1	0.125	0.25	0.25	2	0.063
Yersinia pseudotuberculosis	ATCC 29833	0.5	0.25	0.063	≤0.031	0.5	0.25	0.25	>32	2	≤0.031
Gram-positive bacteria											
Bacillus subtilis	ATCC 6633	32	4	1	0.063	0.25	4	2	8	0.25	≤0.031
Enterococcus faecalis	ATCC 29212	>32	>32	16	2	2	>32	32	>32	>32	1
Lactobacillus casei	ATCC 393	>32	4	32	1	0.5	4	2	>32	2	1
Micrococcus luteus	ATCC 9341	4	0.5	≤0.031	0.063	≤0.031	0.5	0.5	>32	1	2
Staphylococcus aureus	ATCC 29213	32	8	8	0.125	1	8	32	>32	2	0.5
Streptococcus pneumoniae	ATCC 49619	2	0.5	≤0.031	0.063	0.5	0.5	0.25	>32	32	0.5
Streptococcus pyogenes	ATCC 10389	1	0.125	≤0.031	≤0.031	0.063	0.125	0.125	>32	16	0.125

a CTX-M type β-lactamase producer.

b Resistant to azithromycin.

c Resistant to ceftriaxone.

d VIM-10 and VEB-1 β-lactamase producer.

e Resistant to tetracycline and trimethoprim-sulfamethoxazole.

f MICs of cefiderocol (CFDC) were determined in iron-depleted cation-adjusted Mueller Hinton broth (ID-CAMHB), and those of other antibiotics were determined in CAMHB except when the following was used (see supplemental method 2): (i) CAMHB supplemented with 2.5 to 5.0% lysed horse blood for B. parapertussis, Streptococcus pneumoniae, Streptococcus pyogenes, Campylobacter jejuni, Neisseria meningitidis, and Lactobacillus casei, (ii) Haemophilus test medium broth for Haemophilus spp., or (iii) GC agar supplemented with 1% defined growth supplement for Neisseria gonorrhoeae, except for meropenem, which was determined on GC agar supplemented with 1% defined growth supplement without cysteine component. NT, not tested; CFDC, cefiderocol; CAZ, ceftazidime; CFPM, cefepime; MEPM, meropenem; PIPC-TAZ, piperacillin-tazobactam; CAZ-AVI, ceftazidime-avibactam; CFT-TAZ, ceftolozane-tazobactam; COL, colistin; AMK, amikacin; CPFX, ciprofloxacin. TAZ and AVI were at a fixed concentration of 4 μg/ml. MICs of PIPC-TAZ, CAZ-AVI, and CFT-TAZ are represented as the concentrations of PIPC, CAZ, and CFT, respectively.

**TABLE 2:** MICs of cefiderocol and other antibiotics against anaerobic bacteria

Organism	Strain	MIC (μg/ml)^*a*^				
		Cefiderocol	Cefepime	Meropenem	Ciprofloxacin	Metronidazole
Gram-negative bacteria						
Bacteroides fragilis	ATCC 25285	2	32	0.063	2	0.25
Bacteroides thetaiotaomicron	ATCC 29741	>32	>32	0.125	16	1
Fusobacterium mortiferum	ATCC 25557	>32	>32	0.25	2	0.5
Fusobacterium necrophorum	ATCC 25286	≤0.031	≤0.031	≤0.031	1	0.125
Mobiluncus curtisii	ATCC 35241	1	0.25	≤0.031	0.5	2
Prevotella bivia	ATCC 29303	>32	>32	0.25	32	1
Prevotella intermedia	ATCC 25611	1	0.25	≤0.031	0.5	0.5
Prevotella melaninogenica	ATCC 25845	2	0.25	≤0.031	1	0.5
Veillonella parvula	ATCC 10790	32	1	0.125	0.125	2
Gram-positive bacteria						
Bifidobacterium bifidum	ATCC 29521	0.5	0.063	≤0.031	8	4
Clostridium difficile	ATCC 700057	>32	>32	1	8	0.125
Clostridium perfringens	ATCC 13124	1	0.25	≤0.031	0.25	0.5
Collinsella aerofaciens	ATCC 25986	>32	2	0.125	1	0.5
Eubacterium limosum	ATCC 8486	0.5	≤0.031	≤0.031	1	0.125
Finegoldia magna	ATCC 29328	8	8	0.063	0.25	0.5
Parvimonas micra	ATCC 33270	1	0.125	≤0.031	0.5	0.5
Peptoniphilus asaccharolyticus	ATCC 14963	32	0.063	≤0.031	0.5	0.5
Peptostreptococcus anaerobius	ATCC 27337	8	0.5	0.25	1	0.25
Propionibacterium acnes	ATCC 6919	8	0.5	≤0.031	0.5	>32

a MICs were determined on brucella agar supplemented with hemin, vitamin K1, and laked sheep blood.

**TABLE 3:** MIC_50_s and MIC_90_s of cefiderocol and other antibiotics against anaerobic bacteria

Organism (no. of strains)^*a*^	MIC_50_/MIC_90_ (μg/ml)^*b*^				
	Cefiderocol	Cefepime	Meropenem	Ciprofloxacin	Metronidazole
Bacteroides spp. (83)	>32/>32	>32/>32	0.125/2	16/>32	0.5/1
Prevotella spp. (37)	32/>32	4/>32	≤0.031/0.125	1/>32	0.5/2
C. difficile (38)	>32/>32	>32/>32	1/2	8/>32	0.125/0.125

a Bacteriodes spp. consisted of *B. caccae* (4 strains), B. fragilis (52), B. fragilis group (9), *B. ovatus* (2), *B. stercoris* (1), B. thetaiotaomicron (11), and B. vulgatus (4). Prevotella spp. consisted of *P. bivia* (11 strains), *P. buccae* (7), *P. disiens* (4), P. intermedia (10), P. melaninogenica (1), *P. oralis* (1), and Prevotella species (6).

b MICs were determined on brucella agar supplemented with hemin, vitamin K1, and laked sheep blood.

### Antibacterial activity against Gram-negative bacteria harboring various β-lactamases.

Cefiderocol exhibited potent *in vitro* activity against 33 strains of Gram-negative bacteria harboring various kinds of β-lactamases, including extended-spectrum β-lactamases (ESBLs) and carbapenemases (Table 4). The MICs of cefiderocol were 8 μg/ml or lower against all the test strains, including carbapenemase producers such as Klebsiella pneumoniae harboring NDM-1, KPC, or GES-4, P. aeruginosa harboring VIM or IMP-1, and A. baumannii harboring OXA-23 and/or OXA-51-like or OXA-58. Other tested antibiotics, including various classes of β-lactams, amikacin, and ciprofloxacin, showed less activity against most of these carbapenemase producers, with MICs of 16 μg/ml or more, while colistin showed MICs of 1 μg/ml or lower.

**TABLE 4:** MICs of cefiderocol against Gram-negative bacteria harboring β-lactamases

Organism	Strain	β-Lactamase(s)	MIC (μg/ml)^*a*^									
			CFDC	CAZ	CFPM	MEPM	PIPC-TAZ	CAZ-AVI	CFT-TAZ	COL	AMK	CPFX
E. coli	SR34250	CTX-M-14, TEM-1	0.125	2	4	≤0.031	2	0.25	0.5	0.25	2	32
E. coli	SR34201	CTX-M-15, TEM-1	2	>32	>32	0.063	2	0.25	0.5	0.5	2	32
E. coli	SR34241	CTX-M-27	1	8	8	≤0.031	2	0.25	0.5	0.25	2	>32
E. coli	ATCC BAA-196	TEM-10	1	>32	4	≤0.031	8	2	2	0.25	8	0.25
E. coli	ATCC BAA-198	TEM-26	0.5	>32	4	≤0.031	4	1	1	0.25	2	0.25
K. pneumoniae	ATCC 51983	SHV-5	0.5	>32	2	0.063	2	0.25	1	0.5	2	≤0.031
K. pneumoniae	ATCC 700603	SHV-18	1	32	0.5	0.063	16	0.5	1	0.25	1	0.5
K. pneumoniae	NUBL-KG502	GES-4	0.25	>32	16	16	32	16	>32	0.25	32	0.063
P. aeruginosa	SR24837	PER-1	4	>32	>32	8	>32	>32	>32	0.5	8	1
K. pneumoniae	VA-360	KPC-2, TEM-1, SHV-11, SHV-12	8	>32	>32	>32	>32	2	>32	0.25	16	>32
K. pneumoniae	VA-375	KPC-3, TEM-1, SHV-11, SHV-14	2	>32	32	32	>32	2	>32	0.25	8	>32
E. coli	NUBL-24	IMP-1	1	>32	>32	8	16	>32	>32	0.25	2	>32
P. aeruginosa	SR27060	IMP-1	0.25	>32	>32	>32	>32	>32	>32	1	16	32
A. baumannii	SBRKM-181	IMP-1	0.125	>32	>32	32	32	>32	>32	0.5	16	0.5
K. pneumoniae	SR08933	IMP-6	0.125	>32	>32	32	4	>32	>32	0.5	1	32
E. coli	IR5	NDM-1, CTX-M-15, OXA-9	2	>32	>32	>32	>32	>32	>32	0.25	>32	>32
K. pneumoniae	I1	NDM-1, SHV-12	2	>32	>32	32	>32	>32	>32	8	>32	>32
K. pneumoniae	KI2	NDM-1, OXA-1, CTX-M-15, CMY-6, TEM-1, SHV-28, OXA-9	4	>32	>32	>32	>32	>32	>32	0.25	>32	>32
P. aeruginosa	AK54	VIM-2	0.125	32	16	>32	>32	32	>32	1	>32	16
P. aeruginosa	DM3355	VIM-6	2	>32	>32	>32	>32	>32	>32	1	>32	>32
P. aeruginosa	P0510	VIM-1	0.5	32	32	>32	>32	32	>32	1	>32	16
E. coli	SR09616	CMY-2	0.125	>32	1	0.063	32	0.25	4	0.5	2	0.25
K. pneumoniae	NUBL-HKY327	CMY-19	1	>32	>32	0.063	>32	>32	>32	0.25	16	≤0.031
K. pneumoniae	SR09603	CMY-8	0.063	8	0.125	0.25	8	0.25	0.5	0.25	8	≤0.031
K. pneumoniae	SR09635	DHA	0.125	>32	0.125	0.125	>32	0.25	1	0.25	0.5	0.5
S. marcescens	SR36500	AmpC	0.125	8	1	0.25	16	2	8	>32	16	4
P. aeruginosa	TESS	AmpC	0.25	32	16	16	>32	4	2	0.5	>32	32
A. baumannii	585	OXA-23	0.063	>32	32	>32	>32	8	16	0.25	>32	>32
A. baumannii	CHAR	OXA-58	1	>32	32	16	>32	>32	>32	16	>32	>32
A. baumannii	NCTC 13303	OXA-26, OXA-51-like	0.5	>32	>32	>32	>32	>32	>32	0.5	>32	>32
A. baumannii	NCTC 13422	OXA-51-like	0.5	>32	>32	8	>32	>32	>32	0.5	>32	>32
A. baumannii	NCTC 13424	OXA-23, OXA-51-like	≤0.031	>32	32	32	>32	32	32	0.5	>32	>32
K. pneumoniae	PLE	OXA-48	≤0.031	1	2	2	>32	0.25	1	0.25	2	>32

a MICs of cefiderocol were determined in iron-depleted cation-adjusted Mueller Hinton broth (ID-CAMHB), and those of other antibiotics were determined in CAMHB. CFDC, cefiderocol; CAZ, ceftazidime; CFPM, cefepime; MEPM, meropenem; PIPC-TAZ, piperacillin-tazobactam; CAZ-AVI, ceftazidime-avibactam; CFT-TAZ, ceftolozane-tazobactam; COL, colistin; AMK, amikacin; CPFX, ciprofloxacin. TAZ and AVI were at a fixed concentration of 4 μg/ml. MICs of PIPC-TAZ, CAZ-AVI, and CFT-TAZ are represented as the concentrations of PIPC, CAZ and CFT, respectively.

### Affinity for penicillin-binding proteins, morphological changes, and time-kill.

**FIG 1:**
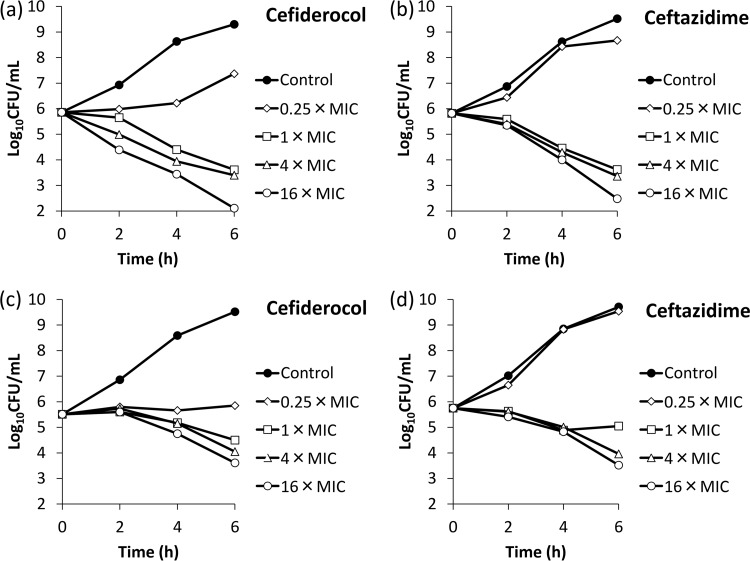
Bactericidal activities of cefiderocol (a and c) and ceftazidime (b and d) against E. coli NIHJ JC-2 (a and b) and K. pneumoniae SR22291 (c and d). ID-CAMHB and CAMHB were used for cefiderocol and ceftazidime, respectively, as the test media.

**FIG 2:**
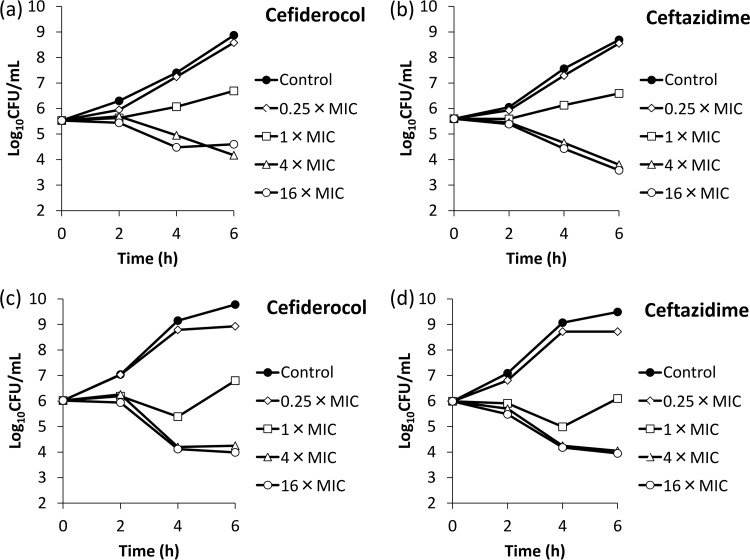
Bactericidal activities of cefiderocol (a and c) and ceftazidime (b and d) against P. aeruginosa ATCC 27853 (a and b) and A. baumannii ATCC 17978 (c and d). ID-CAMHB and CAMHB were used for cefiderocol and ceftazidime, respectively, as the test media.

The affinities (50% inhibitory concentrations [IC_50_s]) of cefiderocol for PBPs of E. coli NIHJ JC-2, K. pneumoniae SR22291, P. aeruginosa ATCC 27853, and A. baumannii ATCC 17978 were determined (Table 5). The IC_50_s of cefiderocol against PBP3 of E. coli NIHJ JC-2, K. pneumoniae SR22291, P. aeruginosa ATCC 27853, and A. baumannii ATCC 17978 were 0.04, 0.062, 0.06, and 0.67 μg/ml, respectively, which were lower than those of ceftazidime, indicating a higher affinity of cefiderocol for PBP3 than that of ceftazidime. Other than its affinity for PBP3, cefiderocol had an affinity for PBP2 of K. pneumoniae SR22291 as ceftazidime did (IC_50_s of cefiderocol and ceftazidime were 0.063 and 0.41 μg/ml, respectively), and cefiderocol also had an affinity for PBP1a of P. aeruginosa ATCC 27853 as ceftazidime did (IC_50_s of cefiderocol and ceftazidime were 0.85 and 3.62 μg/ml, respectively). Morphological changes of these four bacteria were examined by phase-contrast microscopy after exposure to cefiderocol (see Fig. S1 and S2 in the supplemental material). Filamentous cells were observed in all the test strains after exposure to cefiderocol, similar to what was observed after exposure to ceftazidime. In the time-kill study with the four strains E. coli NIHJ JC-2, K. pneumoniae SR22291, P. aeruginosa ATCC 27853, and A. baumannii ATCC 17978, cefiderocol reduced the bacterial counts in a manner similar to that of ceftazidime after exposure at 1, 4, or 16 times the MIC, and the killing rates were similar between 4 times and 16 times the MIC (Fig. 1 and 2).

**TABLE 5:** Affinity of cefiderocol and ceftazidime for penicillin-binding proteins of E. coli NIHJ JC-2, K. pneumoniae SR22291, P. aeruginosa ATCC 27853, and A. baumannii ATCC 17978

PBP	IC_50_ (μg/ml)							
	E. coli NIHJ JC-2		K. pneumoniae SR22291		P. aeruginosa ATCC 27853		A. baumannii ATCC 17978 ^*a*^	
	Cefiderocol	Ceftazidime	Cefiderocol	Ceftazidime	Cefiderocol	Ceftazidime	Cefiderocol	Ceftazidime
PBP1a	3.80	>4	2.80	1.50	0.85	3.62	1.05	0.91
PBP1b	3.37	>4	3.50	2.30	>4	>4		
PBP2	2.12	>4	0.063	0.41	>4	>4	2.31	>64
PBP3	0.04	0.45	0.062	0.22	0.06	0.09	0.67	1.78
PBP4	NC^*b*^	>1	0.28	3.60	>4	>4	ND^*c*^	ND

a The IC_50_s cannot be divided into separate values for PBP1a and PBP1b.

b NC, not calculated. Cefiderocol inhibited 63% of PBP4 at 4 μg/ml, but the sigmoid curve did not fit and the IC_50_ cannot be calculated.

c ND, not detected.

### Effects of transposon insertion into or deletions of genes relating to outer membrane permeability on the activities.

The effects of the deficiency of iron transporters were examined using P. aeruginosa and E. coli (Table 6 and 7). The MICs of cefiderocol against all the test P. aeruginosa PAO1 derivative mutant strains which have a transposon (Tn) insertion in the genes of iron transporters, including major siderophore receptors for pyoverdine (*fpvA* and *fpvB*), pyochelin (*fptA*), and enterobactin (*pirA*), ranged from 0.063 to 0.125 μg/ml, equivalent to the MIC against the parent strain PAO1, with the exception of the MICs against the strains having a Tn insertion in the probable iron transport receptor gene *piuA* (Table 6). The MIC of cefiderocol increased to 2 μg/ml (PW8599) after Tn insertion into *piuA*, which was complemented by the introduction of wild-type PiuA (SR-L00252). MICs of ceftazidime ranged from 1 to 2 μg/ml against all the tested strains with a Tn insertion in iron transporter-related genes as well as the parent strain PAO1. The MIC of cefiderocol against E. coli BW25113 with deletion of iron transporter gene *cirA* or *fiu* was 0.063 or 0.125 μg/ml, within 2-fold of that against the parent strain, whereas the MIC of cefiderocol increased 16-fold by the double knockout of *cirA* and *fiu* (Table 7).

**TABLE 6:** Effects of transposon insertion into genes for iron transporters, efflux pump, and porin on the activities of cefiderocol against P. aeruginosa PAO1

Strain	Description^*a*^	MIC (μg/ml)^*b*^				
		Cefiderocol	Ceftazidime	Imipenem	Ciprofloxacin	Aztreonam
PAO1		0.125	2	1	0.125	4
PW1781	*mexB* (Tn)	0.063	1	1	0.063	0.25
PW1783	*oprM* (Tn)	0.063	1	1	≤0.031	0.25
PW1776	*mexR* (Tn)	0.25	8	1	0.5	16
PW7066	*nalD* (Tn)	0.25	8	1	0.5	16
PW2742	*oprD* (Tn)	0.25	1	8	0.25	4
PW1861	*fiuA* (Tn)	0.063	2	—	—	—
PW2689	*pirA* (Tn)	0.063	2	—	—	—
PW3399	*pfuA* (Tn)	0.063	2	—	—	—
PW4366	*feuA* (*cirA*) (Tn)	0.063	2	—	—	—
PW5036	*fpvA* (Tn)	0.063	2	—	—	—
PW5144	*foxA* (*optS*) (Tn)	0.125	2	—	—	—
PW5503	*pfeA* (Tn)	0.063	1	—	—	—
PW7590	*fecA* (Tn)	0.063	1	—	—	—
PW8065	*fpvB* (Tn)	0.063	2	—	—	—
PW8161	*fptA* (Tn)	0.125	2	—	—	—
PW8599	*piuA* (Tn)	2	2	—	—	—
SR-L00016	*piuA* (Tn) and *pirA* (deletion)	2	2	—	—	—
SR-L00197	*piuA* (Tn)/pMMB67HE-Gm	2	1	—	—	—
SR-L00252	*piuA* (Tn)/pMMB67HE-Gm-piuA	0.063	1	—	—	—

a Tn, transposon insertion.

b —, not tested. MICs of cefiderocol were determined in ID-CAMHB, and those of references were determined in CAMHB.

**TABLE 7:** Effects of deletions of genes for iron transporters and porins on the activities of cefiderocol against E. coli BW25113 and K. pneumoniae NVT2001S

Strain	Description	MIC (μg/ml)^*a*^		
		Cefiderocol	Ceftazidime	Meropenem
E. coli				
BW25113	Derivative strain of K-12	0.063	0.25	
BW25113 *ΔcirA*	*cirA* deletion strain of BW25113	0.063	0.25	
BW25113 *Δfiu*	*fiu* deletion strain of BW25113	0.125	0.125	
BW25113 *ΔcirA Δfiu*	*cirA* and *fiu* deletion strain of BW25113	1	0.25	
K. pneumoniae				
NVT2001S	Streptomycin-resistant isolate of clinical strain NVT2001	0.031	0.125	0.016
NVT2001S *ΔompK35*	*ompK35* deletion strain of NVT2001S	0.125	0.5	0.031
NVT2001S *ΔompK36*	*ompK36* deletion strain of NVT2001S	0.063	0.25	0.031
NVT2001S *ΔompK35/36*	*ompK35* and *ompK36* deletion strain of NVT2001S	0.063	0.5	0.125

a MICs of cefiderocol were determined in ID-CAMHB, and those of references were determined in CAMHB.

The MICs of cefiderocol against PAO1 derivative mutant strains which have a Tn insertion in the genes of multidrug efflux pump MexAB-OprM, its transcriptional regulator, and porin OprD, which are involved in β-lactam resistance, were examined (Table 6). MICs of aztreonam against the strains with a Tn insertion in either *mexB* (PW1781) or *oprM* (PW1783), which lost the function of the MexAB-OprM efflux pump, were 16-fold lower than that against PAO1, while the decreases in cefiderocol MICs due to a Tn insertion were 2- or 4-fold. The MIC of cefiderocol was also determined against PW1781 and PW1783 in cation-adjusted Mueller-Hinton broth (CAMHB), which contains ferric iron, and the cefiderocol MICs were 0.125 and 0.063 μg/ml, respectively, which were 2- or 4-fold lower, respectively, than that against PAO1, which was 0.25 in CAMHB. The MICs of ceftazidime, aztreonam, and ciprofloxacin against the strains with a Tn insertion in either *mexR* (PW1776) or *nalD* (PW7066), which leads to overexpression of the MexAB-OprM efflux pump, were 4-fold higher than that against PAO1, while the increases in cefiderocol MICs due to a Tn insertion were within 2-fold. The MIC of cefiderocol was also determined against PW1776 and PW7066 in CAMHB, and the increases in cefiderocol MICs were also 2-fold compared to that against PAO1. Against the strain with a Tn insertion in *oprD*, imipenem showed an 8-fold higher MIC than against the parent strain, while the increases in MIC of cefiderocol and other test antibiotics were within 2-fold.

The MIC of cefiderocol was determined against K. pneumoniae NVT2001S and its derivative mutant strains, which were deficient in porin *ompK35* and/or *ompK36*, which are involved in resistance to various classes of β-lactam antibiotics, including carbapenems (Table 7). The MIC of meropenem against the double deletion mutant strain was 8-fold higher than that against the parental strain NVT2001S, whereas the increases in MICs of cefiderocol and ceftazidime against strains with deletion of *ompK35* and/or *ompK36* were 2- to 4-fold that against the parental strain.

## DISCUSSION

In comparison to carbapenem antibiotics, cefiderocol has more potent *in vitro* activity against a broad range of Gram-negative bacteria, including carbapenem-resistant strains of Enterobacteriaceae and nonfermenting bacteria that produce carbapenemases as well as ESBLs and class C β-lactamases, but weaker activity against Gram-positive bacteria and anaerobic bacteria. Cefiderocol has an antibacterial spectrum that is significantly different from that of carbapenem antibiotics, which have been mainly used for the treatment of Gram-negative bacterial infections. The reason for this unique antibacterial spectrum is the active ferric iron uptake by the siderophore, which is observed only in aerobic Gram-negative bacteria. The detailed mode of action on the activity against some of the strains or species among Gram-positives and anaerobic bacteria has not been sufficiently observed. The global surveillance studies of cefiderocol, SIDERO-WT-2014, using recent clinical isolates (*n* = 9,205) against Enterobacteriaceae and nonfermenting bacteria such as P. aeruginosa, A. baumannii, and S. maltophilia, including MDR strains, and other studies to evaluate the antibacterial activity of cefiderocol against well-characterized carbapenem-resistant Gram-negative pathogens have shown that cefiderocol also has potent activity against these problematic Gram-negative pathogens (10–12).

This study revealed that the antibacterial action of cefiderocol is to inhibit mainly PBP3 of Enterobacteriaceae and nonfermenting bacteria, resulting in morphological changes of filamentous cells, similar to the action of ceftazidime. Although the impact of the differences in PBP3 affinity on the *in vitro* activities of cefiderocol and ceftazidime is not clear, the antibacterial activity of cefiderocol determined by the time-kill experiment was similar to that of ceftazidime. The key features of cefiderocol are its active uptake mechanisms by Gram-negative bacteria under iron-depleted conditions and its improved stability to various types of β-lactamases reported previously (15). In this study, we showed that PiuA is one of the iron transporters of P. aeruginosa that is responsible for the active transport of cefiderocol into bacterial cells resulting in the activity of cefiderocol, as is the case for MC-1 (16). However, the MIC results also showed that PirA, which was reported to be one of the iron transporters responsible for the activity of MC-1 (16), did not contribute to the *in vitro* activity of cefiderocol. Moreover, this study demonstrated the contribution of both *cirA* and *fiu* of E. coli, which has been reported to be involved in the monomeric catechols and whose production is regulated by the availability of ferric iron, to the *in vitro* activity of cefiderocol (17).

The MIC results with mutant strains showed that the effect of the deficiency of the porin OmpK35/36 of K. pneumoniae, which is reported to be one of the resistance determinants coordinated with various β-lactamases against carbapenems (18, 19), on the activity of cefiderocol was not significant, which may also contribute to the potent activity of cefiderocol against such carbapenem-resistant K. pneumoniae strains. In terms of the efflux pump MexAB-OprM of P. aeruginosa, the decrease in the MIC of cefiderocol against the strains with a deficient efflux pump indicates that cefiderocol could be a substrate for the efflux pump MexAB-OprM. However, the overproduction of the efflux pump increased the cefiderocol MIC only slightly, even under the condition with ferric iron in the medium, in which the function of active transport for cefiderocol is weak; this indicates that the effect of the overproduction on the activity of cefiderocol should be limited and that cefiderocol is not taken into bacterial cells faster than it is extruded from the bacterial cells by the efflux pump. On the other hand, it has been reported that BAL30072 is a substrate for the efflux pumps MexAB-OprM and MexEF-OprN (20) and that MC-1 is a substrate for the efflux pump MexAB-OprM (16). Those reports suggest that cefiderocol has different profiles of transport into P. aeruginosa with other siderophore-conjugated β-lactams. These studies are limited in clarifying and understanding the differences in the mechanisms of action of cefiderocol and other siderophore-conjugated β-lactams, and further detailed studies are required.

In summary, this study showed that cefiderocol has potent *in vitro* activity against a broad range of aerobic Gram-negative bacteria, including MDR strains, and that the antibacterial activity of cefiderocol is based mainly on the inhibition of PBP3. This study also revealed that iron transporters such as PiuA of P. aeruginosa and CirA and Fiu of E. coli are involved in the permeation of cefiderocol into bacterial cells. The characteristics of cefiderocol indicate that cefiderocol may be a promising option for the treatment of infections caused by a broad range of Gram-negative pathogens, including MDR Enterobacteriaceae and MDR nonfermenting bacteria.

## MATERIALS AND METHODS

### Bacterial strains.

A number of type strains were obtained from the American Type Culture Collection (Manassa, VA), the National Collection of Type Cultures (Salisbury, United Kingdom), and the National Institute of Technology and Evaluation Biological Resource Center (Tokyo, Japan). A number of clinical strains were kindly provided by the Bicêtre Hospital (Le Kremlin-Bicêtre, France), Singapore General Hospital (Singapore), and GlaxoSmithKline plc (Middlesex, United Kingdom). Other test strains were obtained from various hospitals, mainly in Japan. Species-appropriate quality control (QC) strains, which were obtained from the ATCC, were used as described in Clinical and Laboratory Standards Institute (CLSI) guidelines (21–24). Transposon (Tn) insertion mutant strains of P. aeruginosa PAO1 were kindly provided by the University of Washington (25). A PAO1 derivative of SR-L00016 was constructed from PW8599 by deletion of the *pirA* gene according to the method described by Alexeyev et al. and Schweizer et al. (26, 27). The PiuA expression plasmid was constructed by using an In-Fusion HD cloning kit (TaKaRa Bio, Inc., Shiga, Japan) with pMMB67HE-Gm, and PW8599 was transformed with the plasmid to obtain SR-L00252. The *cirA* and/or *fiu* deletion mutants of E. coli BW25113 were constructed according to the methods described by Datsenko et al. (28). Detailed procedures for constructing the plasmid and recombinant strains are described in the supplemental material (see Method S1). K. pneumoniae NVT2001S and its *ompK35* and/or *ompK36* deletion mutants were kindly provided by the National Health Research Institutes in Taiwan.

### Antibiotics.

Cefiderocol, ceftolozane, and avibactam were synthesized at the research laboratories of Shionogi & Co., Ltd. (Osaka, Japan). Commercial-grade antibiotics were obtained as follows: ceftazidime, tazobactam, amikacin, and aztreonam from Chem-Impex International, Inc. (Wood Dale, IL); cefepime and metronidazole from U.S. Pharmacopeia (Rockville, MD); meropenem, colistin, and gentamicin from Wako Pure Chemical Industries, Ltd. (Osaka, Japan); and ciprofloxacin and piperacillin from LKT Laboratories, Inc. (St. Paul, MN).

### MIC.

MICs were determined by using broth microdilution or agar dilution according to the CLSI (21–24), except for the MIC for Bordetella parapertussis, which was determined by the method described by Mortensen and Rodgers (29). For the determination of cefiderocol MIC, iron-depleted cation-adjusted Mueller-Hinton broth (ID-CAMHB) was prepared as previously described and used according to the recommendations of the CLSI (30), except for the cases that are required to determine MICs under specific conditions (Method S2). The quality control MIC ranges of cefiderocol approved by the CLSI were 0.06 to 0.5 μg/ml for both E. coli ATCC 25922 and P. aeruginosa ATCC 27853 (31). For anaerobic bacteria, brucella agar (Becton, Dickinson and Company, NJ) supplemented with hemin, vitamin K1, and laked sheep blood was used. For recombinant strains, test medium was supplemented with 10 μg/ml of gentamicin and/or 0.1 mM IPTG (isopropyl-β-d-thiogalactopyranoside) (Wako Pure Chemical Industries) when required.

### Affinity for penicillin-binding proteins.

The affinities of cefiderocol and ceftazidime for PBPs of E. coli NHIJ JC-2, K. pneumoniae SR22291, and P. aeruginosa ATCC 27853 were determined by using benzylpenicillin [benzyl-^14^C]potassium (American Radiolabeled Chemicals, Inc., St. Louis, MO) as described by Spratt (32). For A. baumannii ATCC 17978, the method using Bocillin FL penicillin sodium salt (Life Technologies, Inc., Carlsbad, CA) reported by Vashist et al. (33) was used. Detailed procedures are described in the supplemental material (Method S3).

### Morphological observation.

A bacterial suspension of the log-phase bacteria was smeared on compound-containing thin-layer Mueller-Hinton agar (Becton, Dickinson and Company, NJ) coated on a glass slide. After incubation at 35°C for 4 to 6 h, morphological changes of bacterial cells were observed with Leica DM2500 microscopy (Leica, Germany). Detailed procedures are described in the supplemental material (Method S4).

### Time-kill study.

An overnight culture of the test strain was diluted into fresh medium to yield an inoculum of approximately 10^6^ CFU/ml. The ID-CAMHB and CAMHB media were used for cefiderocol and ceftazidime, respectively. Concentrations of antibiotics were 0 (control), 0.25, 1, 4, or 16 times the MIC. Incubation was performed at 35°C, and the sampling times were 2, 4, and 6 h after initiation of incubation. MICs against E. coli NIHJ JC-2, K. pneumoniae SR22291, P. aeruginosa ATCC 27853, and A. baumannii ATCC 17978 were 0.25, 0.008, 0.063 and 0.016 μg/ml, respectively, for cefiderocol (ID-CAMHB) and 0.25, 0.063, 1 and 4 μg/ml, respectively, for ceftazidime (CAMHB).

## Supplementary Material

Supplemental material

## References

[1] Kanj SS, Whitelaw A, Dowzicky MJ 2014 *In vitro* activity of tigecycline and comparators against Gram-positive and Gram-negative isolates collected from the Middle East and Africa between 2004 and 2011. Int J Antimicrob Agents 43:170–178. doi:10.1016/j.ijantimicag.2013.10.011. 24315313

[2] WHO. 2017 WHO publishes list of bacteria for which new antibiotics are urgently needed. WHO, Geneva, Switzerland http://www.who.int/mediacentre/news/releases/2017/bacteria-antibiotics-needed/en/.

[3] Tängdén T, Giske CG 2015 Global dissemination of extensively drug-resistant carbapenemase-producing Enterobacteriaceae: clinical perspectives on detection, treatment and infection control. J Intern Med 277:501–512. doi:10.1111/joim.12342. 25556628

[4] Foley T, Simeonov A 2012 Targeting iron assimilation to develop new antibacterials. Expert Opin Drug Discov 7:831–847. doi:10.1517/17460441.2012.708335. 22812521 PMC3434712

[5] Mollmann U, Heinisch L, Bauernfeind A, Kohler T, Ankel-Fuchs D 2009 Siderophores as drug delivery agents: application of the “Trojan Horse” strategy. Biometals 22:615–624. doi:10.1007/s10534-009-9219-2. 19214755

[6] Wencewicz TA, Miller MJ 2017 Sideromycins as pathogen-targeted antibiotics. In Topics in medicinal chemistry. Springer, Berlin, Germany.

[7] Kim A, Kutschke A, Ehmann DE, Patey SA, Crandon J, Gorseth E, Miller AA, McLaughlin RE, Blinn CM, Chen A, Nayar AS, Dangel B, Tsai AS, Rooney MT, Murphy-Benenato KE, Eakin AE, Nicolau DP 2015 Pharmacodynamic profiling of a siderophore-conjugated monocarbam in *Pseudomonas aeruginosa*: assessing the risk for resistance and attenuated efficacy. Antimicrob Agents Chemother 59:7743–7752. doi:10.1128/AAC.00831-15. 26438502 PMC4649189

[8] Tomaras AP, Crandon JL, McPherson CJ, Banevicius MA, Finegan SM, Irvine RL, Brown MF, O'Donnell JP, Nicolau DP 2013 Adaptation-based resistance to siderophore-conjugated antibacterial agents by *Pseudomonas aeruginosa*. Antimicrob Agents Chemother 57:4197–4207. doi:10.1128/AAC.00629-13. 23774440 PMC3754284

[9] Tomaras AP, Crandon JL, McPherson CJ, Nicolau DP 2015 Potentiation of antibacterial activity of the MB-1 siderophore-monobactam conjugate using an efflux pump inhibitor. Antimicrob Agents Chemother 59:2439–2442. doi:10.1128/AAC.04172-14. 25605364 PMC4356814

[10] Dobias J, Dénervaud-Tendon V, Poirel L, Nordmann P 26 7 2017 Activity of the novel siderophore cephalosporin cefiderocol against multidrug-resistant Gram-negative pathogens. Eur J Clin Microbiol Infect Dis. doi:10.1007/s10096-017-3063-z. 28748397

[11] Falagas ME, Skalidis T, Vardakas KZ, Legakis NJ 2017 Activity of cefiderocol (S-649266) against carbapenem-resistant Gram-negative bacteria collected from inpatients in Greek hospitals. J Antimicrob Chemother 72:1704–1708. doi:10.1093/jac/dkx049. 28369471

[12] Hackel MA, Tsuji M, Yamano Y, Echols R, Karlowsky JA, Sahm DF 2017 *In vitro* activity of the siderophore cephalosporin, cefiderocol, against a recent collection of clinically relevant Gram-negative bacilli from North America and Europe, including carbapenem-nonsusceptible isolates (SIDERO-WT-2014 Study). Antimicrob Agents Chemother 61:e00093-17. doi:10.1128/AAC.00093-17. 28630181 PMC5571285

[13] Ito A, Kohira N, Bouchillon SK, West J, Rittenhouse S, Sader HS, Rhomberg PR, Jones RN, Yoshizawa H, Nakamura R, Tsuji M, Yamano Y. 2016a In vitro antimicrobial activity of S-649266, a catechol substituted siderophore cephalosporin, when tested against non-fermenting gram-negative bacteria. J Antimicrob Chemother 71:670–677. 26645269 10.1093/jac/dkv402

[14] Kohira N, West J, Ito A, Ito-Horiyama T, Nakamura R, Sato T, Rittenhouse S, Tsuji M, Yamano Y 2016 *In vitro* antimicrobial activity of a siderophore cephalosporin, S-649266, against *Enterobacteriaceae* clinical isolates, including carbapenem-resistant strains. Antimicrob Agents Chemother 60:729–734. doi:10.1128/AAC.01695-15. 26574013 PMC4750680

[15] Ito-Horiyama T, Ishii Y, Ito A, Sato T, Nakamura R, Fukuhara N, Tsuji M, Yamano Y, Yamaguchi K, Tateda K 2016 Stability of novel siderophore cephalosporin S-649266 against clinically relevant carbapenemases. Antimicrob Agents Chemother 60:4384–4386. doi:10.1128/AAC.03098-15. 27139465 PMC4914688

[16] McPherson CJ, Aschenbrenner LM, Lacey BM, Fahnoe KC, Lemmon MM, Finegan SM, Tadakamalla B, O'Donnell JP, Mueller JP, Tomaras AP 2012 Clinically relevant Gram-negative resistance mechanisms have no effect on the efficacy of MC-1, a novel siderophore-conjugated monocarbam. Antimicrob Agents Chemother 56:6334–6342. doi:10.1128/AAC.01345-12. 23027195 PMC3497185

[17] Nikaido H, Rosenberg EY 1990 Cir and Fiu proteins in the outer membrane of *Escherichia coli* catalyze transport of monomeric catechols: study with beta-lactam antibiotics containing catechol and analogous groups. J Bacteriol 172:1361–1367. doi:10.1128/jb.172.3.1361-1367.1990. 2407721 PMC208606

[18] Tsai Y-K, Fung C-P, Lin J-C, Chen J-H, Chang F-Y, Chen T-L, Siu LK 2011 *Klebsiella pneumoniae* outer membrane porins OmpK35 and OmpK36 play roles in both antimicrobial resistance and virulence. Antimicrob Agents Chemother 55:1485–1493. doi:10.1128/AAC.01275-10. 21282452 PMC3067157

[19] Tsai Y-K, Liou C-H, Fung C-P, Lin J-C, Siu LK 2013 Single or in combination antimicrobial resistance mechanisms of *Klebsiella pneumoniae* contribute to varied susceptibility to different carbapenems. PLoS One 8:e79640. doi:10.1371/journal.pone.0079640. 24265784 PMC3827147

[20] Page MG, Dantier C, Desarbre E 2010 *In vitro* properties of BAL30072, a novel siderophore sulfactam with activity against multiresistant gram-negative bacilli. Antimicrob Agents Chemother 54:2291–2302. doi:10.1128/AAC.01525-09. 20308379 PMC2876421

[21] Clinical and Laboratory Standards Institute. 2015 Methods for dilution antimicrobial susceptibility tests for bacteria that grow aerobically, approved standard M7-A10, 7th ed Clinical and Laboratory Standards Institute, Wayne, PA.

[22] Clinical and Laboratory Standards Institute. 2012 Methods for antimicrobial susceptibility testing of anaerobic bacteria, approved standard M11-A8, 8th ed Clinical and Laboratory Standards Institute, Wayne, PA.

[23] Clinical and Laboratory Standards Institute. 2015 Methods for antimicrobial dilution and disk susceptibility testing of infrequently isolated or fastidious bacteria, approved standard M45-A3, 3rd ed Clinical and Laboratory Standards Institute, Wayne, PA. 10.1086/510431 17173232

[24] Clinical and Laboratory Standards Institute. 2016 Performance standards for antimicrobial susceptibility testing, 26th informational supplement, M100-S26 Clinical and Laboratory Standards Institute, Wayne, PA.

[25] Jacobs MA, Alwood A, Thaipisuttikul I, Spencer D, Haugen E, Ernst S, Will O, Kaul R, Raymond C, Levy R, Chun-Rong L, Guenthner D, Bovee D, Olson MB, Manoil C 2003 Comprehensive transposon mutant library of *Pseudomonas aeruginosa*. Proc Natl Acad Sci U S A 100:14339–14344. doi:10.1073/pnas.2036282100. 14617778 PMC283593

[26] Alexeyev MF, Shokolenko IN, Croughan TP 1995 Improved antibiotic-resistance gene cassettes and omega elements for *Escherichia coli* vector construction and *in vitro* deletion/insertion mutagenesis. Gene 160:63–67. doi:10.1016/0378-1119(95)00108-I. 7628718

[27] Schweizer HP, Hoang TT 1995 An improved system for gene replacement and *xylE* fusion analysis in *Pseudomonas aeruginosa*. Gene 158:15–22. doi:10.1016/0378-1119(95)00055-B. 7789804

[28] Datsenko KA, Wanner BL 2000 One-step inactivation of chromosomal genes in Escherichia coli K-12 using PCR products. Proc Natl Acad Sci U S A 97:6640–6645. doi:10.1073/pnas.120163297. 10829079 PMC18686

[29] Mortensen JE, and Rodgers GL 2000 In vitro activity of gemifloxacin and other antimicrobial agents against isolates of Bordetella pertussis and Bordetella parapertussis. J Antimicrob Chemother 45(Suppl S1):47–49. doi:10.1093/jac/45.suppl_3.47. 10824032

[30] Ito A, Nishikawa T, Matsumoto S, Yoshizawa H, Sato T, Nakamura R, Tsuji M, Yamano Y 2016 Siderophore cephalosporin cefiderocol utilizes ferric iron transporter systems for antibacterial activity against *Pseudomonas aeruginosa*. Antimicrob Agents Chemother 60:7396–7401. doi:10.1128/AAC.01405-16. 27736756 PMC5119021

[31] Huband MD, Ito A, Tsuji M, Sader HS, Fedler KA, Flamm RK 2017 Cefiderocol MIC quality control ranges in iron-depleted cation-adjusted Mueller-Hinton broth using a CLSI M23-A4 multi-laboratory study design. Diagn Microbiol Infect Dis 88:198–200. doi:10.1016/j.diagmicrobio.2017.03.011. 28410852

[32] Spratt BG 1977 Properties of the penicillin-binding proteins of *Escherichia coli* K12. Eur J Biochem 72:341–352. doi:10.1111/j.1432-1033.1977.tb11258.x. 319999

[33] Vashist J, Tiwari V, Das R, Kapil A, Rajeswari MR 2011 Analysis of penicillin-binding proteins (PBPs) in carbapenem resistant *Acinetobacter baumannii*. Indian J Med Res 133:332–338. 21441690 PMC3103161

[34] Ghazi IM, Monogue ML, Tsuji M, Nicolau DP 2017 Pharmacodynamics of cefiderocol, a novel siderophore cephalosporin, explored in a *Pseudomonas aeruginosa* neutropneic murine thigh model. Int J Antimicrob Agents doi:10.1016/j.ijantimicag.2017.10.008. 29111435

