# Supplementary material for: In Vitro Antibacterial Properties of Cefiderocol, a Novel Siderophore Cephalosporin, against Gram-Negative Bacteria
Source: Antimicrob Agents Chemother. 2017 Dec 21;62(1):e01454-17. doi: 10.1128/AAC.01454-17 (PMC5740388; doi:10.1128/AAC.01454-17)
Supplement: Supplemental material [file supp_62_1_e01454-17__index.html]

In Vitro Antibacterial Properties of Cefiderocol, a Novel Siderophore Cephalosporin, against Gram-Negative Bacteria — Supplemental material 

# *In Vitro* Antibacterial Properties of Cefiderocol, a Novel Siderophore Cephalosporin, against Gram-Negative Bacteria

## Supplemental material

- Supplemental file 1 -

  Supplemental text, Fig. S1 and S2

  PDF, 1.3M
