# Supplementary material for: In Vitro Antibacterial Properties of Cefiderocol, a Novel Siderophore Cephalosporin, against Gram-Negative Bacteria
Source: Antimicrob Agents Chemother. 2017 Dec 21;62(1):e01454-17. doi: 10.1128/AAC.01454-17 (PMC5740388; doi:10.1128/AAC.01454-17)
Supplement: Supplemental material [file AAC.01454-17_zac001186779s1.pdf]

**Figure S1. Observation of morphological change in the presence of 1×MIC of cefiderocol or ceftazidime**

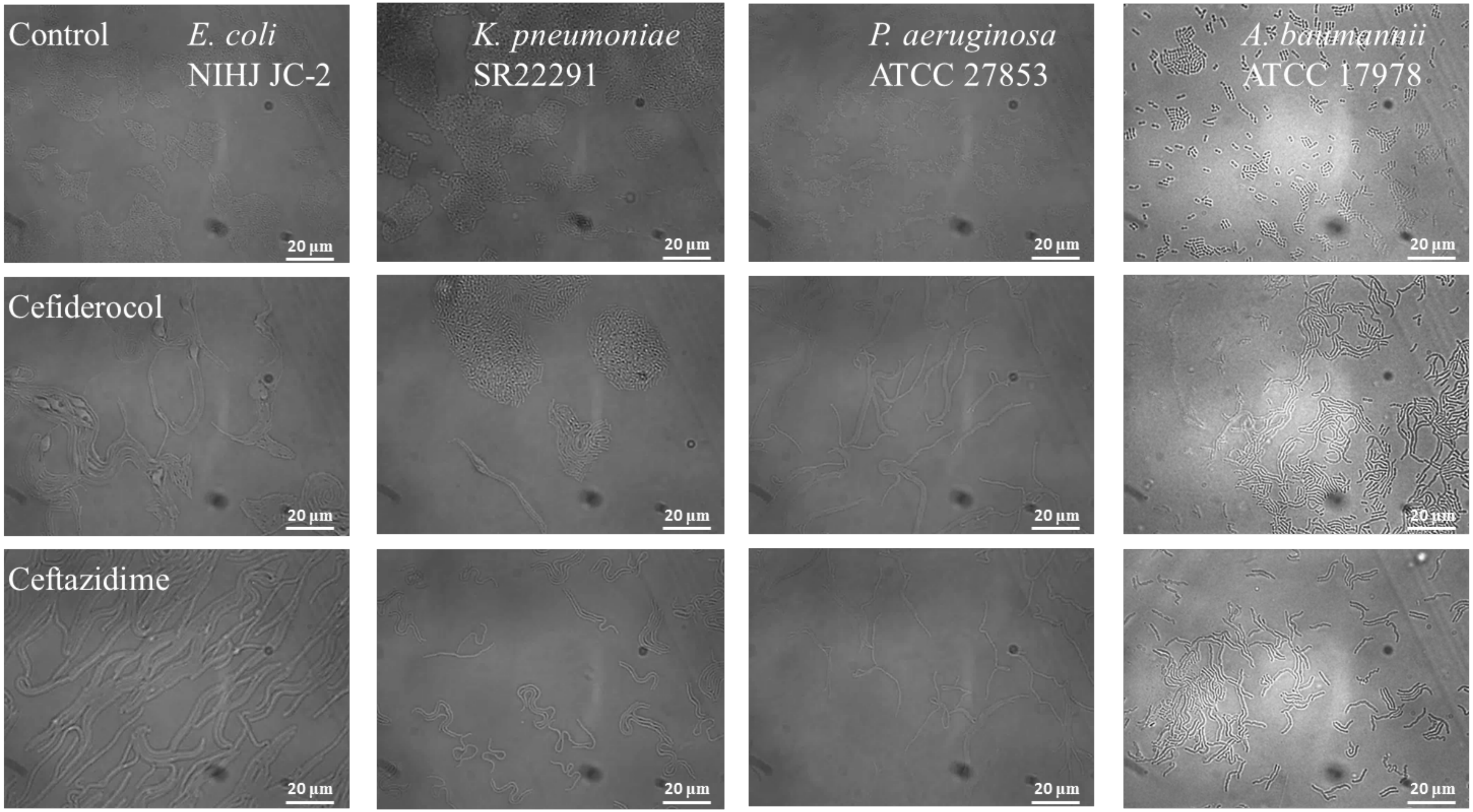

**Figure S2. Observation of morphological change in the presence of 4×MIC of cefiderocol or ceftazidime**

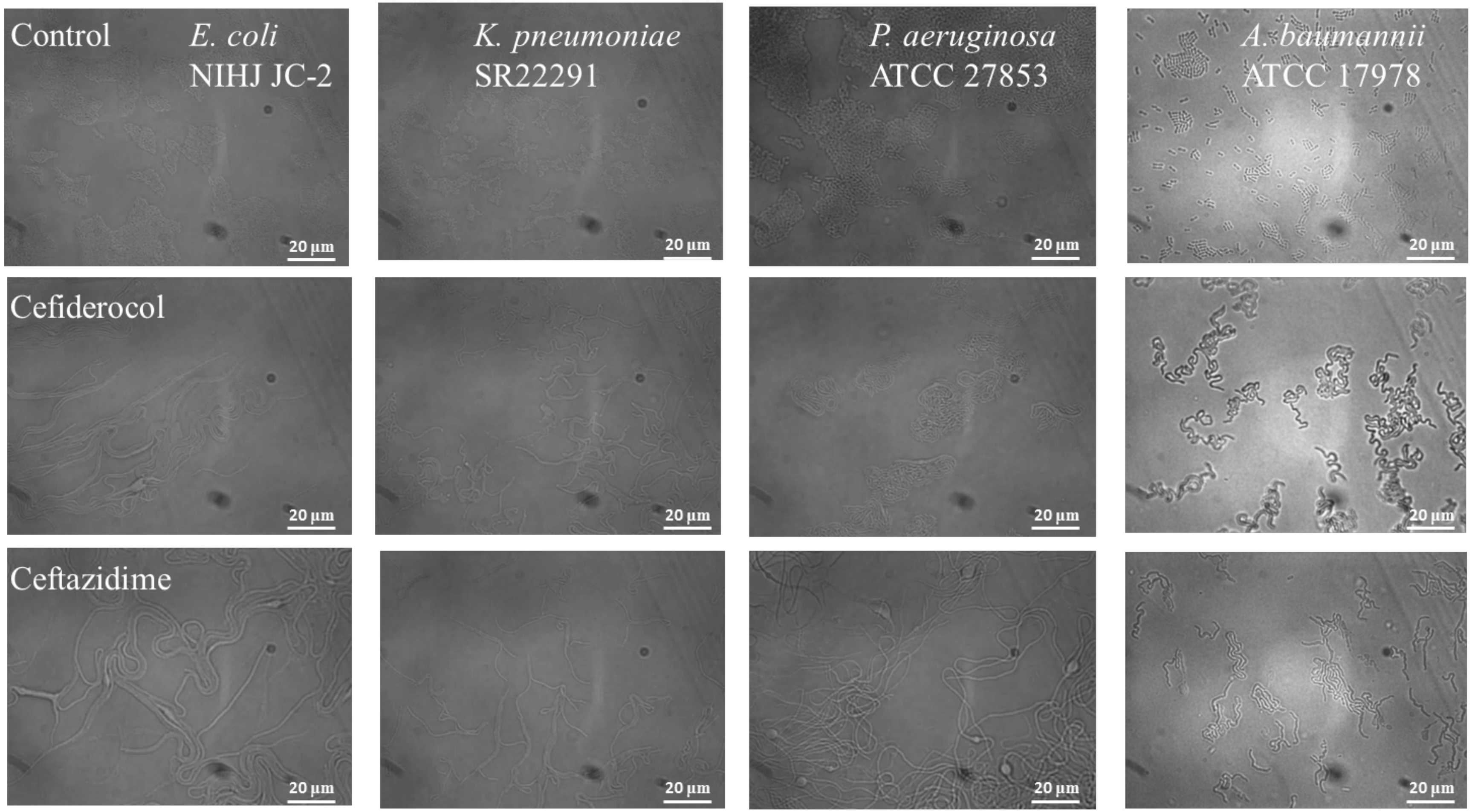

**Supplemental method 1. Construction of deletion mutant strains**

The strain of *P. aeruginosa* SR-L00016 was constructed from PW8599 with the deletion of *pirA* gene following the method described by Alexeyev et al. and Schweizer et al. (1-2). The *pirA* gene fragment amplified by PCR was cloned into NsiI site of pEX100TG (pEX100T::Gm$^{R}$ cassette from pBSL142), followed by removal of the internal EcoNI fragment on *pirA* gene of the plasmid. *E. coli* S17-1 was transformed with the resultant plasmid, and the plasmid was transferred to PW8599 by conjugation to make the homologous recombination on the *pirA* of PW8599. The *pirA* gene deletion was screened and verified by PCR and sequencing.

The PiuA expression plasmid was constructed using pMMB67HE-Gm (replaced ampicillin-resistant cassette of pMMB67HE with gentamycin-resistant cassette). The *piuA* gene fragment was amplified by PCR and cloned into pMMB67HE-Gm by using In-Fusion® HD Cloning Kit (Takara Bio Inc., Shiga, Japan), obtaining pMMB67HE-Gm-PiuA. *P. aeruginosa* PW8599 (PAO1 harboring transposon insertion into *piuA*) was transformed with pMMB67HE-Gm and pMMB67HE-Gm-PiuA to obtain the strains of SR-L00197 and SR-L00252, respectively.

The deletion mutant strains of *E. coli* BW25113 were constructed according to the methods described by Datsenko et al (3). Briefly, a FLT-flanked kanamycin marker was amplified and transformed into strain expressing λ Red recombinase with pKD46. Kanamycin-resistant transformant was selected, and kanamycin cassette was eliminated using FLP expression plasmid pCP20.

| Purpose | Primers (forward/reverse) |
|---|---|
| Deletion of *pirA* | 5’-CCAATGCATGCATGCAGCAACTGGTGGACAACAC-3’<br>5’-CACCGCAATATCCGGTCATGCATGCCGATTC-3’ |
| Cloning of *piuA* | 5’-GGAAACAGAATTAAGCTATGTCGCGTCAGTCCACGGATAC-3’<br>5’-TCGACCTGCAGGCATGCAAGCTTCAGAAGTGGAAGTTCACCCCGAGC-3’ |
| Deletion of *cirA* | 5'-GAGCGATAACCCATTTTATTTTCGTAGTTACCTCATGGAGATATGGAATGATTCCGGGGATCCGTCGACC-3'<br>5'-GTCCGCCCGGTTCGCCGGGCATCTTCTCATCAGAAGCGATAATCCACTGCTGTAGGCTGGAGCTGCTTCG-3' |

| | |
|---|---|
| Deletion of *fiu* | 5'-CTCGTGGCAGTGAAAATTTCAACATATAAGAAAAAGTCACCTGCAAAATGATTCCGGGGATCCGTCGACC-3'<br>5'-AAAAGTGGGGCCTGCGCCCCACATCTGAATCAGAAATGCATATTGGCTGTTGTAGGCTGGAGCTGCTTCG-3' |

**Supplemental method 2. MIC conditions that are used for some species**

CAMHB supplemented with 2.5 to 5.0% of lysed horse blood (LHB) were used and cultured in ambient atmosphere for *B. parapertussis*, *Streptococcus pneumoniae*, and *Streptococcus pyogenes*, in microaerophilic atmosphere for *Campylobacter jejuni*, and in 5% $CO_2$ atmosphere for *Neisseria meningitidis* and *Lactobacillus casei*. MIC against *Haemophilus* spp. was determined in Haemophilus Test Medium (HTM) broth (Nissui Pharmaceutical Co., Ltd., Tokyo, Japan). MIC against *Neisseria gonorrhoeae* was determined on GC agar (Hardy Diagnostics, Santa Maria, CA) supplemented with 1% defined growth supplement in 5% $CO_2$ atmosphere except for meropenem which was determined on GC agar supplemented with 1% defined growth supplement without cysteine component.

For anaerobic bacteria, Brucella agar (Becton, Dickinson and Company, NJ) supplemented with hemin (Wako Pure Chemical Industries, Ltd., Osaka, Japan), Vitamin K1 (Wako Pure Chemical Industries, Ltd., Osaka, Japan), and laked sheep blood (Nippon Bio-test laboratories Inc., Saitama, Japan) was used. For recombinant strains, test medium was supplemented with 10 μg/mL of gentamicin and/or 0.1 mM of isopropyl β-D-1-thiogalactopyranoside (IPTG) (Wako Pure Chemical Industries) when required.

**Supplemental method 3. PBP affinity**

The affinity of cefiderocol and ceftazidime for PBPs of *E. coli* NHIJ JC-2, *K. pneumoniae* SR22291 and *P. aeruginosa* ATCC 27853 were determined as described by Spratt et al. (Spratt). Briefly, the membrane fraction was incubated with antibiotics, and benzylpenicillin [benzyl-$^{14}$C] potassium ([14C]-PCG, final concentration was 30 to 40 μg/mL) (American Radiolabeled Chemicals, Inc., Saint Louis, MO) was added and incubated. Samples were applied to SDS-PAGE to separate PBPs, and the labeled PBPs were detected and analyzed by STORM (Amersham Bioscience Corp., Buckinghamshire, UK). $IC_{50}$, which was defined as the concentration required for inhibiting 50% of the binding of [$^{14}$C]-PCG to PBPs, of antibiotic against each PBP was determined using WinNonlin (Pharsight, CERTARA, CA).

The affinity of cefiderocol and ceftazidime for PBPs of *A. baumannii* ATCC 17978 was determined as described by Vashist et al (Vashist). Briefly, the membrane fraction was incubated with antibiotics, and Bocillin™ FL penicillin sodium salt (Boc-PCV, final concentration was 100 μg/mL) (Life Technologies, Inc., Carlsbad, CA) was added and incubated. Samples were applied to SDS-PAGE to separate PBPs, and the florescent density of the labeled PBPs was scanned using an Alexa 555 application of VersaDoc 5000 MP gel scanner (BIO-RAD) and Quantity One (BIO-RAD). $IC_{50}$ of antibiotic was calculated by fitting sigmoidal dose-response model using XLfit (version 5.3.1.3) (Guildford, Surrey, UK).

**Supplemental method 4. Observation of morphological change**

Morphological changes of *E. coli* NIHJ JC-2, *K. pneumoniae* SR22291, *P. aeruginosa* ATCC 27853, and *A. baumannii* ATCC 17978 in the presence of 1×MIC or 4×MIC of cefiderocol or ceftazidime. Bacterial suspension in CAMHB was incubated with constant shaking at 35°C. The log-phase bacterial cells were smeared on compound-containing thin-layer Mueller Hinton agar (MHA; MHB; Becton, Dickinson and Company, NJ) that was coated on glass slide, and then incubated again at 35°C. Bacterial cells were incubated 4 to 6 h with or without the concentration corresponding to MIC, and morphological changes of bacterial cells were observed with microscopy Leica DM2500 (Leica, Germany). The MICs were determined on MHA by agar dilution method, and those against *E. coli* NIHJ JC-2, *K. pneumoniae* SR22291, *P. aeruginosa* ATCC 27853, and *A. baumannii* ATCC 17978 were 0.25, 0.004, 0.25 and 0.25 µg/mL, respectively, for cefiderocol and 0.25, 0.063, 2 and 4 µg/mL, respectively, for ceftazidime.

## References


1. **Alexeyev MF, Shokolenko IN, Croughan TP.** 1995. Improved antibiotic-resistance gene cassettes and omega elements for *Escherichia coli* vector construction and *in vitro* deletion/insertion mutagenesis. Gene. **160:**63-67.
2. **Schweizer HP, Hoang TT.** 1995. An improved system for gene replacement and *xylE* fusion analysis in *Pseudomonas aeruginosa*. Gene **158:**15–22.
3. **Datsenko KA, Wanner BL.** 2000. One-step inactivation of chromosomal genes in Escherichia coli K-12 using PCR products. Proc Natl Acad Sci USA **97:**6640-6645.
